# An Unusual Member of the Papain Superfamily: Mapping the Catalytic Cleft of the *Marasmius oreades* agglutinin (MOA) with a Caspase Inhibitor

**DOI:** 10.1371/journal.pone.0149407

**Published:** 2016-02-22

**Authors:** Gabriele Cordara, André van Eerde, Elin M. Grahn, Harry C. Winter, Irwin J. Goldstein, Ute Krengel

**Affiliations:** 1 Department of Chemistry, University of Oslo, Oslo, Norway; 2 Department of Biochemistry, Institute for Cancer Research, The Norwegian Radium Hospital, Oslo, Norway; 3 Department of Biological Chemistry, Medical School, University of Michigan, Ann Arbor, Michigan, United States of America; Russian Academy of Sciences, Institute for Biological Instrumentation, RUSSIAN FEDERATION

## Abstract

Papain-like cysteine proteases (PLCPs) constitute the largest group of thiol-based protein degrading enzymes and are characterized by a highly conserved fold. They are found in bacteria, viruses, plants and animals and involved in a number of physiological and pathological processes, parasitic infections and host defense, making them interesting targets for drug design. The *Marasmius oreades* agglutinin (MOA) is a blood group B-specific fungal chimerolectin with calcium-dependent proteolytic activity. The proteolytic domain of MOA presents a unique structural arrangement, yet mimicking the main structural elements in known PLCPs. Here we present the X-ray crystal structure of MOA in complex with Z-VAD-fmk, an irreversible caspase inhibitor known to cross-react with PLCPs. The structural data allow modeling of the substrate binding geometry and mapping of the fundamental enzyme-substrate interactions. The new information consolidates MOA as a new, yet strongly atypical member of the papain superfamily. The reported complex is the first published structure of a PLCP in complex with the well characterized caspase inhibitor Z-VAD-fmk.

## Introduction

Cysteine proteases are involved in a number of physiological and pathological processes. They are classified in families based on sequence and fold conservation and further grouped into superfamilies or clans [1]. There are currently 77 recognized families of cysteine proteases in the MEROPS database, divided into 13 clans of C (Cys) and P (mixed) types, 48 of which have been structurally characterized [2].

**Fig 1:**
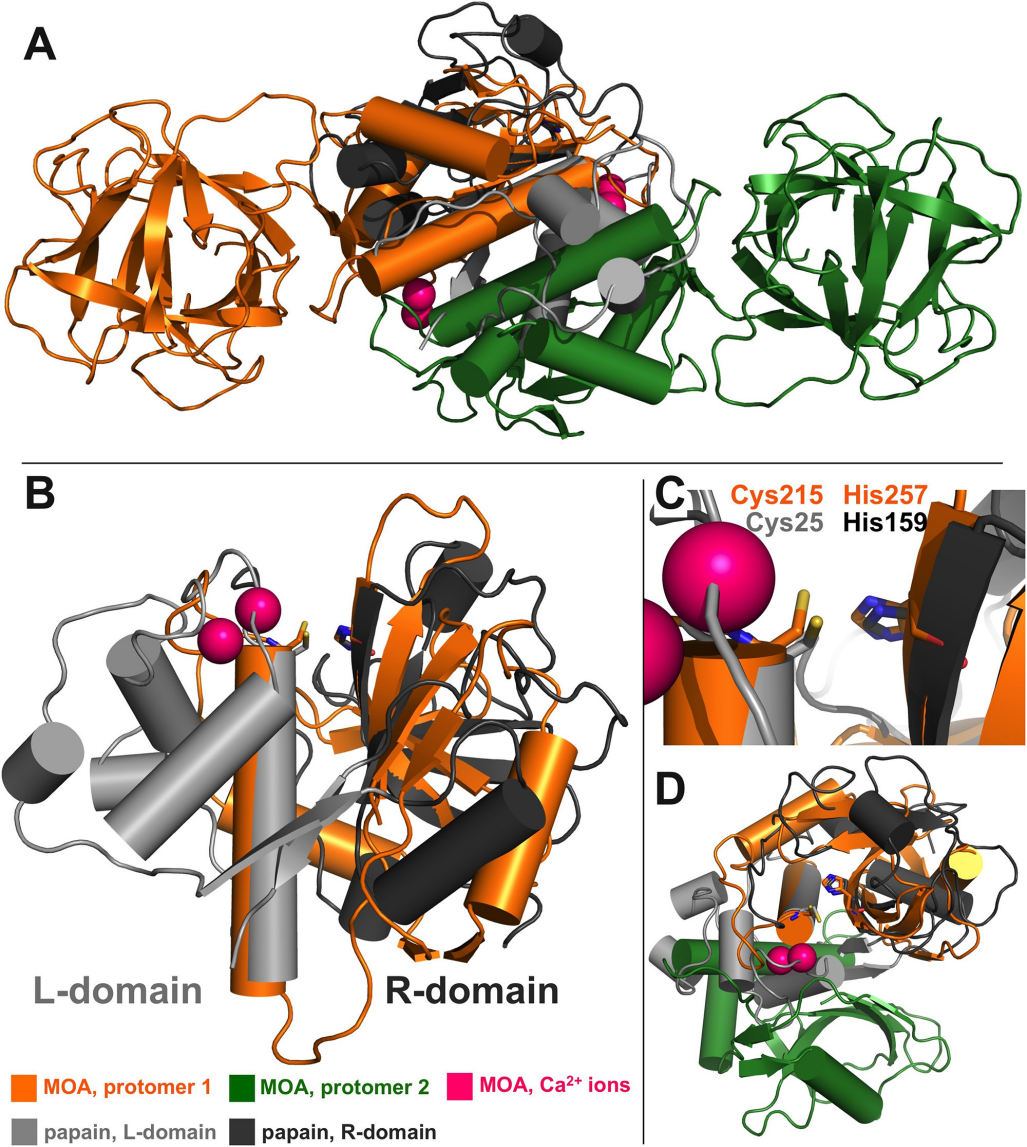
Comparison of MOA and papain PLCP domains. (A) The dimerization domain of MOA is a good structural match of papain and papain-like cysteine proteases. (B,C) This is clearly visible from the structural superposition of MOA (PDB ID: 3EF2 [14]) and papain (PDB ID: 1CVZ [15]), aligned according to the *standard view*, first described by Heinemann *et al*. [16]. The catalytic Cys-His dyads are indicated. In the figure, the L(eft)- and R(ight)-domain of papain are represented in different colors. (D) The fold conservation between the two enzymes is partially lost in the L-domain, where most structural elements of papain are replaced by the MOA dimerization interface.

Proteases belonging to clan CA are referred to as *papain-like cysteine proteases* (PLCPs, EC 3.4.22) and take their namesake from papain, the superfamily holotype. All PLCPs have the same fold, composed of two subdomains, the L(left)- and R(right)-domain, named after their position in the standard view (Fig 1). They feature a Cys-His catalytic dyad and a conserved enzyme-substrate interaction geometry [3, 4]. These enzymes constitute the cysteine protease superfamily with the largest number of members [5]. PLCPs are found in bacteria, viruses, plants, and animals [6]. They are involved in a number of physiological and pathological processes, including antigen presentation [6], cancer, inherited diseases, parasitic infections [7] and host defense [8, 9]. Their role in pathology makes them suitable targets for drug design [10]; moreover, some of the enzymes can be exploited in integrated pest management [9, 11]. PLCPs are also represented among the fungal taxa. While recognized members include homologues of animal proteases such as bleomycin hydrolase [8], deubiquitinating enzymes [12] and calpain [13], little is known about fungal-specific families of proteases carrying the papain fold.

The *Marasmius oreades* agglutinin (MOA) is a 293 amino acid, homodimeric, histo-blood-group-B specific chimerolectin extracted from the fruiting bodies of the common fairy ring mushroom [17]. Recent literature suggests a role for MOA and related proteins as active players in fungal defense against external threats [18–20]. Each MOA protomer is composed of two domains, accounting for the lectin’s sugar-binding and proteolytic functions, respectively [21]. The proteolytic activity is associated to the C- terminal α+β dimerization domain, which closely resembles the consensus papain fold (r.m.s.d.: 2.2 Å), including the Cys/His catalytic dyad (Cys215, His257) (Fig 1) [18, 22]. The papain-like L- and R-domain partitioning is conserved across the MOA dimer, with the L-domain borrowing structural elements from the other protomer. In contrast to other known PLCPs, the proteolytic domain of MOA carries a binuclear calcium binding site [14]. Calcium binding leads to an active site rearrangement essential for catalysis [14, 18, 22].

X-ray crystal structures of PLCP-inhibitor complexes have historically been fundamental to gain a better understanding of the active site structure, the enzyme-substrate interaction geometry and the subtle differences determining substrate specificity [23]. As with other cysteine-dependent enzymes, the proteolytic activity of MOA can be inhibited by thiol-modifying agents (*e*.*g*., *N*-ethyl maleimide, iodoacetamide) and by specific thiol-reactive compounds.

**Fig 2:**
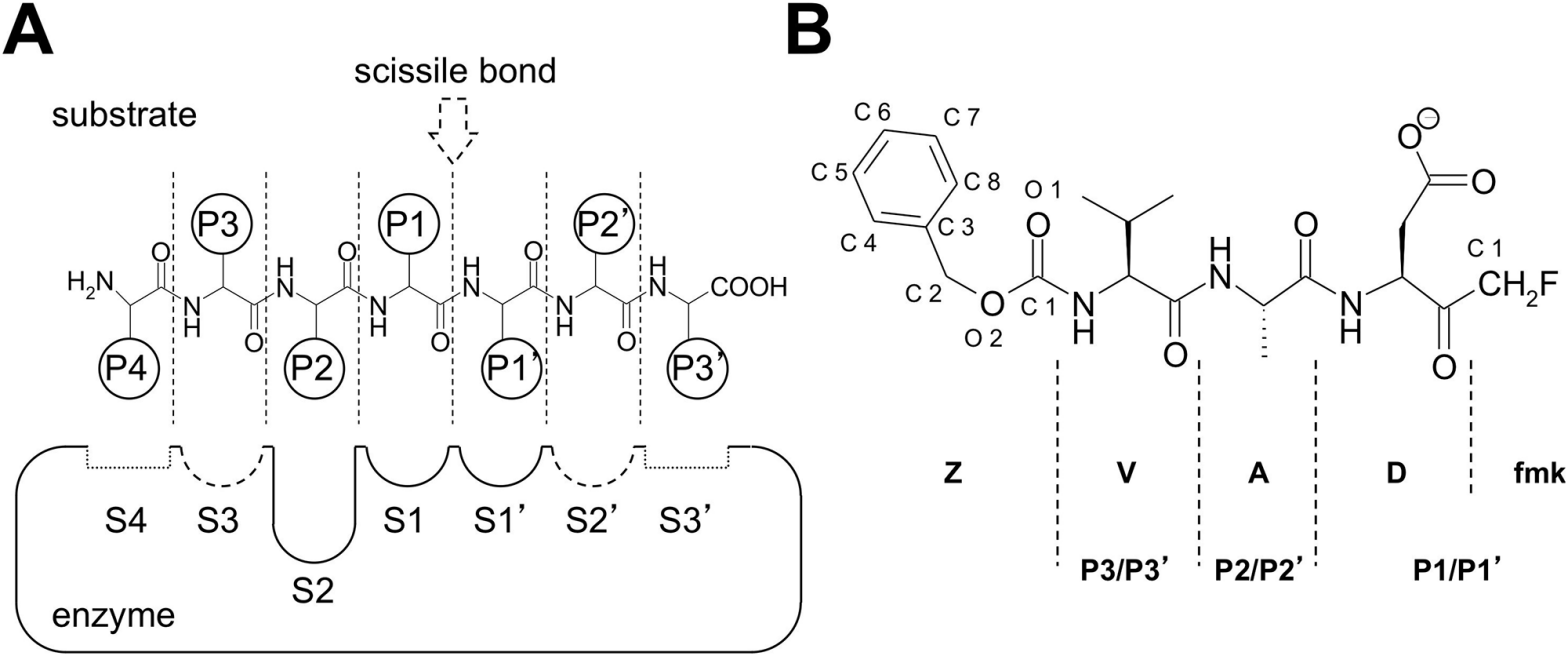
Z-VAD-fmk inhibitor and interaction with PLCP active site. (A) PLCP active site, as mapped by Schechter & Berger [32] and revised by Turk *et al*. [4]; figure adapted from [33]. In a simplified representation of a generic PLCP substrate, the residues on the N-terminal side of the scissile bond are defined as P1-P4 moieties, counting outwards, while those on the C-terminal side are defined as P1’-P3’. Following this description, the scissile bond lies between positions P1 and P1’. The binding subsites on the enzyme are numbered S1-S4 (*unprimed* subsites) and S1’-S3’ (*primed* subsites), depending on the substrate position that they interact with. The S2 binding site is represented as a deeper well to highlight its character of substrate binding pocket. Subsites S3 and S2’ are drawn as dotted lines to represent their nature of “binding areas”. Sites S4 and S3’ are represented as shallow grooves to stress their very low conservation among enzymes of the PLCP superfamily. (B) The Z-VAD-fmk molecule is a substrate-mimetic Val-Ala-Asp tripeptide inhibitor carrying a thiol-reactive fluoromethylketone (fmk) on the carboxy-terminus and a capping benzoxyl carbonyl (Z) moiety on its N-terminus.

Z-VAD-fmk (Fig 2) belongs to a family of irreversible substrate-mimetic ketone inhibitors. Originally designed as an inhibitor for the aspartate-specific ICE/caspase-1 cysteine protease [24, 25], Z-VAD-fmk was subsequently found to efficiently inhibit the proteolytic activity of other enzymes in the caspase family [26]. Later publications have shown cross-reactivity between caspase-specific inhibitors and unrelated thiol-dependent enzymes, including peptide:*N*-glycanases (PNGases) [27] and PLCPs [28], Z-VAD-fmk has been successfully used to assess the physiological role of caspases [29]. In spite of available crystal structures of Z-VAD-fmk in complex with different caspases and a PNGase [30, 31], there is currently no structure of the inhibitor in complex with any representative of the PLCP class of proteases.

The unique features of the PLCP domain of MOA and its nature of fungal-specific papain-like protease provide an opportunity to gain insights into the fungal branch of the papain superfamily. Here, we report X-ray crystal structures of MOA in complex with the Z-VAD-fmk inhibitor. Our work sheds light on the substrate binding geometry at the active site of MOA and identifies the positions of the different binding subsites in the protein’s catalytic cleft. Furthermore, the structural data argue for the correct placement of MOA in the cysteine protease family tree as the representative of a novel fungal-specific PLCP subfamily.

## Materials and Methods

### Expression and purification

An IPTG-inducible pT7 vector (MOApT7-LO) containing the cDNA for wild-type MOA (described in [22]) was expressed in *E*. *coli* strain BL21 (DE3). Bacteria were grown at 37°C in LB medium until the early log phase, induced using 1 mM IPTG and grown at 18°C for 24 hours. Cells were harvested by centrifugation (5000 rcf, 15 min), washed once with a buffer containing 50 mM Tris pH 8.0 and 0.15 M NaCl and stored at -80°C overnight before lysis. After thawing, the bacterial pellets were resuspended in a lysis buffer containing 50 mM Tris pH 8.0, 0.15 M NaCl, 2 mM EDTA, 1x concentrated complete protease inhibitor cocktail EDTA free (Roche Diagnostics Ltd), 1 μl/ml Benzonase nuclease (Thermo Scientific) and 4 mg/ml hen egg white lysozyme. After incubation on a shaker for two hours at RT, the insoluble fraction was removed by two rounds of centrifugation (20000 rcf, 45 min).

The purification protocol for MOA takes advantage of the residual affinity of the sugar binding domain for galactose: as the capture step, the clarified cell lysate was passed through a D-Gal-sepharose affinity column (Thermo Scientific), followed by extensive washing with 20 mM Tris pH 8.0 buffer and elution of the protein using a 1.0 M D-Gal single step gradient. Protein fractions were pooled and concentrated using a 10000 MWCO PES membrane (Vivaspin, Sartorius AG), followed by overnight dialysis against 20 mM acetate pH 4.5, 2 mM EDTA, 2 mM DTT using 7000 MWCO Snakeskin dialysis tubing (Thermo Scientific). Further purification was carried out by cation exchange on a HiTrap SP XL column (GE Healthcare Life Sciences), using a loading buffer containing 20 mM acetate pH 4.5, 2 mM EDTA, 2 mM DTT and eluting the protein with a single step 1.0 M NaCl gradient. Final polishing of the protein preparation was carried out by concentrating the protein to a volume of 500 μl using 10000 MWCO PES membrane concentrator tubes followed by size-exclusion chromatography using a Superdex 200 10/300 GL column (GE Healthcare Life Sciences) and a buffer containing 20 mM acetate pH 5.0, 2 mM EDTA, 0.2 M D-Gal, 0.15 M NaCl and 2 mM DTT. The fractions containing the purified protein were pooled, concentrated to a final protein concentration of 15–20 mg/ml using concentrator tubes with a 10000 MWCO PES membrane (Vivaspin, Sartorius AG) and underwent three rounds of buffer exchange against 20 mM acetate pH 5.0, 2 mM EDTA, 2 mM DTT.

### Crystallization

Before crystallization, MOA proteolytic activity was tested as described in [22]. MOA crystals grew from a solution containing the purified protein at a concentration of 5 mg/ml, pre-mixed directly before the experiments with the Galα1,3(Fucα1,2)Gal trisaccharide (Dextra; 1:20 MOA:sugar molar ratio) and the Z-VAD-fmk inhibitor (Sigma-Aldrich). Z-VAD-direct, Z-VAD-inverted and Z-VAD-dual crystals were obtained from 1:3, 1:20 and 1:30 molar ratios (MOA:inhibitor), respectively. The formulation of the crystallization mixture differed among the three crystal forms with respect to precipitant concentration and absence or the presence of DMSO. The crystallization solutions had the following composition: Z-VAD-direct: 0.1 M imidazole pH 8.0, 12% PEG 8000, 0.2 M calcium acetate; Z-VAD-inverted: 0.1 M imidazole pH 8.0, 10% PEG 8000, 5% DMSO, 0.2 M calcium acetate; Z-VAD-dual: 0.1 M imidazole pH 8.0, 16% PEG 8000, 5% DMSO, 0.2 M calcium acetate. The crystals grew as trigonal prisms with dimensions of 0.1 mm × 0.1 mm × 0.2 mm. Fully grown crystals were cryoprotected in mother liquor supplemented with 15% ethylene glycol and flash-frozen in liquid nitrogen for data collection. The protein consistently crystallized in space group *P*6_3_22, with cell parameters of approximately *a* = 121 Å, *b* = 121 Å, and *c* = 100 Å.

### Data collection, processing, scaling and structure determination

Final diffraction data were collected at beamlines ID29 and ID23-2 at the European Synchrotron Radiation Facility (ESRF, Grenoble, France). The images were processed and scaled using XDS [34]. A summary of the data collection and scaling statistics is given in Table 1. All the structures were solved by molecular replacement with the software PHASER [35], using a modified model of the calcium-bound structure of MOA as search model (PDB ID: 3EF2 [14], lacking the Pro54-Val56 loop and with residues showing flexible or generally poorly defined side chains mutated to Ala). The PHASER solution identified a single MOA protomer in the asymmetric unit; and m*F*o-D*F*c maps showed well-defined, positive electron density peaks for the binuclear metal binding site, the three sugar binding sites and the putative active site cleft.

**Table 1:** Data collection and refinement statistics.

	ZVAD-direct	ZVAD-dual	ZVAD-inverted
**A. Data collection**			
Beamline	ESRF ID29	ESRF ID23-2	ESRF ID23-2
Wavelength (Å)	0.9763	0.8726	0.8726
Collected data (deg.)	45	67	50
Oscillation range (deg.)	0.1	0.4	0.5
Space group	*P*6_3_22	*P*6_3_22	*P*6_3_22
Cell parameters: a, b, c (Å)	120.6 120.6 100.1	121.1 121.1 100.0	121.0 121.0 100.0
Resolution (Å)^a^	46.31–1.60 (1.63–1.60)	46.42–1.65 (1.68–1.65)	46.41–1.70 (1.73–1.70)
*R*_merge_ (%)^a^^b^	6.0 (67.8)	12.1 (71.1)	9.0 (61.0)
*R*_meas_ ^a^^c^	6.7 (76.4)	12.9 (80.6)	10.2 (75.9)
*R*_p.i.m._ ^a^^d^	3.0 (34.4)	4.6 (36.2)	4.6 (43.5)
CC_1/2_	99.9 (77.4)	99.6 (70.3)	99.7 (64.4)
Mean I / σ(I)^a^	15.1 (2.3)	11.2 (1.6)	12.0 (1.4)
Completeness (%)^a^	99.6 (97.1)	93.3 (64.7)	98.5 (83.2)
Multiplicity^a^	4.8 (4.8)	7.2 (4.0)	4.7 (2.6)
No. reflections (unique)	275191 (56827)	355074 (48993)	221657 (47195)
**B. Refinement**			
Resolution (Å)	46.31–1.60	46.42–1.65	46.41–1.70
*R*_work_/*R*_free_ (%)^e^	16.5 / 18.8	16.9 / 19.0	17.7 / 20.3
Macromolecules / a.s.u.	1	1	1
*No*. *atoms*			
Protein	2361	2390	2386
Water	262	277	229
Ligands	130	147	134
*B-factor (Å*^*2*^*)*			
Protein	18.6	13.2	16.8
Water	28.6	24.0	27.0
Ligands	25.3	20.6	26.3
*r*.*m*.*s*.*d*. *from ideal values*			
Bond lengths (Å)	0.02	0.02	0.02
Bond angles (deg.)	2.1	2.0	2.0
*Ramachandran plot*			
Core region (%)	97.4	97.1	97.4
Outliers (%)	0.0	0.0	0.0
PDB ID	5D61	5D62	5D63

^a^Values in parentheses refer to highest resolution shell

^b^*R*_merge_ = Σ_**h**_Σ_*j*_ |*I*_hj_ - 〈*I*_**h**_〉| / Σ_**h**_Σ_*j*_ *I*_hj_, where 〈*I*_**h**_〉 is the mean intensity of symmetry-related reflections *I*_**h**_

^c^*R*_meas_ = Σ_**h**_ [N_**h**_/(N_**h**_-1)]^1/2^Σ_*i*_ |*I*_hj_ - 〈*I*_**h**_〉| / Σ_**h**_Σ_*i*_ *I*_hj_, where N is the redundancy of reflection **h** [36]

^d^*R*_p.i.m._ = Σ_**h**_ [1/(N_**h**_-1)]^1/2^Σ_*j*_ |*I*_hj_ - 〈*I*_**h**_〉| / Σ_**h**_Σ_*j*_ *I*_hj_ [37]

^e^*R*_free_ was calculated from 5% of randomly selected data for each data set

### Model building and refinement

Real space refinement of the PHASER output was carried out using Coot [38] by first removing ill-defined side chains and the loop Ile53-Asn55, and subsequently adding missing structural elements in a step-wise fashion as the quality of the electron density map improved. Model building was alternated with refinement cycles using REFMAC5 [39]. The model was completed by first adding the metal ions, then the sugar ligands, the water molecules and small ligands present either in the mother liquor (chloride ions, DMSO) or the cryoprotecting solution (ethylene glycol). The Z-VAD-fmk inhibitor was modeled at the end of the refinement process, when the difference electron density map allowed the unambiguous tracing of the inhibitor molecule in the MOA active site. Ligand occupancy was determined by minimizing the residual difference electron density for the inhibitor molecule and taking the B-factors of nearby interacting atoms into account.

The final model contains residues 2–293, with no electron density accountable for the first methionine residue, suggesting that it might be cleaved off during protein synthesis. The three sugar binding sites of MOA show a different preference for the anomeric form of the reducing end galactose of the Galα1,3(Fucα1,2)Gal trisaccharide: while the sugar binding α site (residues 20–48) showed full occupancy for β-D-Gal, the β site (residues 72–100) also contains a minor fraction of the α-D-Gal anomer (unmodeled), and the γ site (residues 123–151) exclusively carries the α-D-Gal anomer. The residual density in the catalytic cleft differs among the three data sets, defining the presence of Z-VAD-fmk in two alternative orientations, referred to as ‘direct’ and ‘inverted’. In the ‘ZVAD-direct’ and ‘ZVAD-inverted’ structures, the inhibitor molecule was modeled at full occupancy in one of these two orientations; additional very low electron density for the opposite inhibitor orientation present in the ‘ZVAD-inverted’ data set was not modeled. In the ‘ZVAD-dual’ structure, occupancies for the two inhibitor conformations were refined to 0.6/0.4 (direct/inverted). Validation of the model was carried out using Coot [38], the MolProbity server (http://molprobity.biochem.duke.edu) [40], and phenix.validate [41]; r.m.s.d. values were calculated using the PDBeFold server (http://www.ebi.ac.uk/msd-srv/ssm/) [42]. Refinement statistics are summarized in Table 1. Electron density maps were mostly calculated using the program FFT, which is part of the CCP4 software suite for macromolecular crystallography [43], except for simulated annealing composite OMIT maps (not shown), which were calculated with phenix.refine [44]. All the figures displaying structural data were generated with PyMOL Molecular Graphics System, version 1.5.0.4 (Schrödinger LLC).

## Results and Discussion

### MOA in complex with the Z-VAD-fmk inhibitor

**Fig 3:**
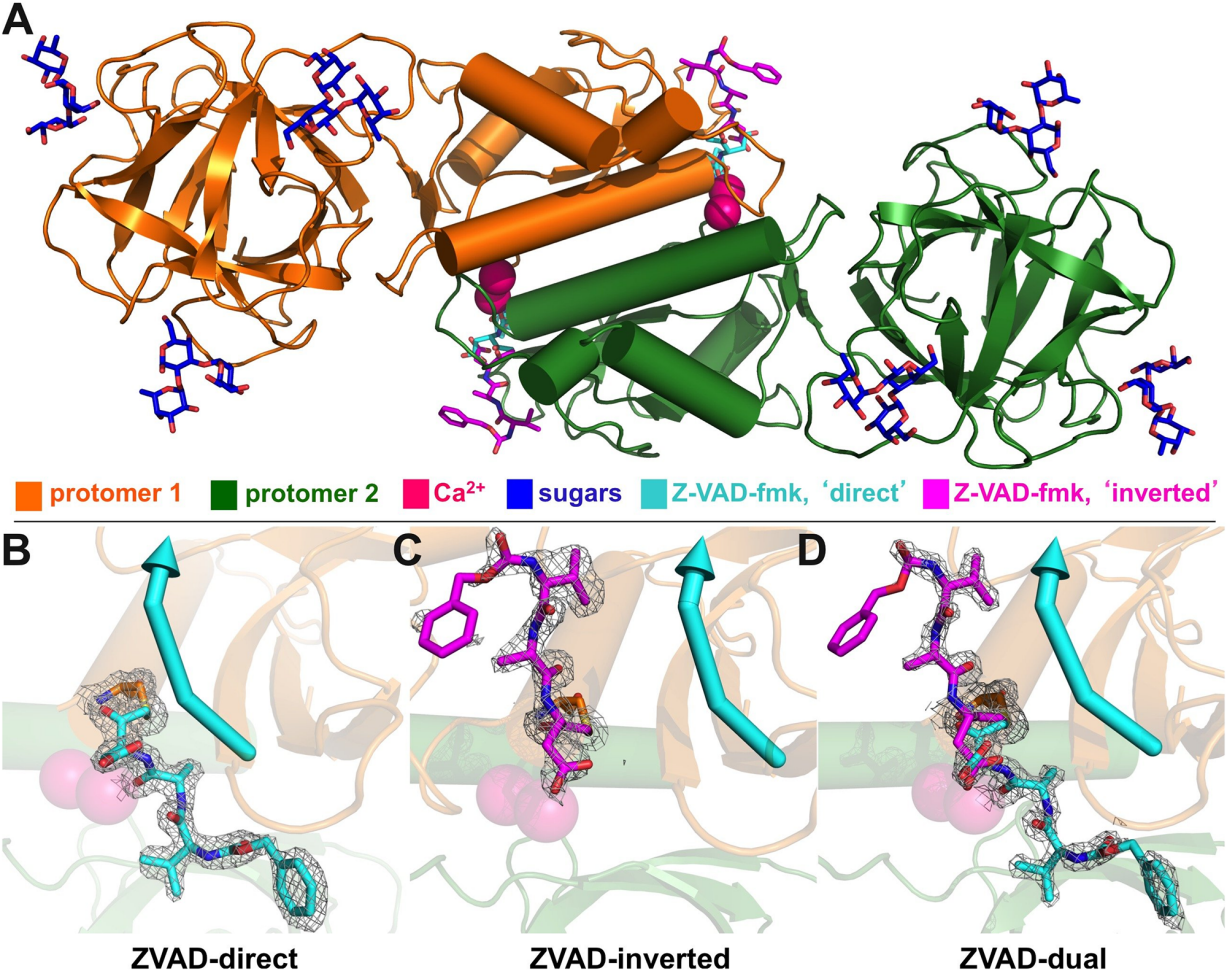
MOA Z-VAD-fmk complex and electron density of the caspase inhibitor. (A) Structure of MOA (‘ZVAD-dual’) in complex with the Z-VAD-fmk inhibitor (cyan/magenta), three blood group B branched trisaccharide ligands (blue) and two calcium ions (dark magenta). The figure includes the symmetry-related protomer (dark green), shown to match the representation of the functional MOA dimer in Fig 1A. The inhibitor molecule was found in two different orientations (B, ‘ZVAD-direct’, cyan; C, ‘ZVAD-inverted’, magenta), interacting with the MOA L- or R-domains, respectively, or in both orientations, with different occupancies (D, ‘ZVAD-dual’); the arrow points towards the C-terminus of a natural PLCP substrate. For all three structures, the figure shows the final σ_A_-weighted 2m*F*o-D*F*c map for Z-VAD-fmk, contoured at 1σ. For stereo figures and electron density before inclusion of the ligand, see S1 Fig.

The structure of MOA in complex with calcium, the irreversible caspase inhibitor Z-VAD-fmk and the branched histo-blood-group B trisaccharide (Galα1,3(Fucβ1,2)Gal) was determined by X-ray crystallography to a resolution of 1.6 Å. In this paper, three different structures are presented, which are based on three independent data sets collected from crystals of the same crystal form (Table 1), which were obtained under slightly different crystallization conditions. The three structures were refined to approximately the same resolution (1.6–1.7 Å), but differ with respect to the orientation and occupancy of the inhibitor, providing unique structural insights into the MOA-Z-VAD-fmk complex. In each case, the structure was independently solved by molecular replacement. The asymmetric unit contains a single MOA protomer, and the biological dimer is generated by crystallographic symmetry (Fig 3A). Apart from the presence of the inhibitor, the structures are essentially identical to the MOA structure without Z-VAD-fmk (r.m.s.d. = 0.1 Å for Cα coordinates), except for minor differences in the side chain orientations for some surface residues.

The MOA Z-VAD-fmk complex provides the very first structural data on the interaction of MOA with a substrate analogue, and thus represents the first attempt at mapping its active site. The three structures reported in the article differ with respect to the residual electron density at the active site, which allowed fitting the Z-VAD-fmk inhibitor molecule in one or two alternative orientations (Fig 3B–3D). The different orientations of Z-VAD-fmk were defined as ‘direct’ or ‘inverted’, depending on whether the VAD peptide was aligned with (‘direct’, Fig 3B) or against (‘inverted’, Fig 3C) the standard backbone orientation of a PLCP substrate [4]. The preferred orientation of the inhibitor molecule seems to correlate with the dimethyl sulfoxide (DMSO) content of the mother liquor, where a higher amount of DMSO seems to favor the presence of the ‘inverted’ orientation. Here, we refer to the structures containing the inhibitor in ‘direct’, ‘inverted’ or both orientations as ‘ZVAD-direct’, ‘ZVAD-inverted’ and ‘ZVAD-dual’, respectively. All structures show well defined electron density from the fluoromethylketone carbon to the valine residue, while the benzyloxycarbonyl tail (Z) remains substantially less well-defined.

The reaction mechanism of thiol-dependent enzymes with halomethylketone derivatives has not yet been fully elucidated, with two alternative reaction routes proposed [45]. In both cases, the nucleophilic attack of a cysteine residue results in the loss of the halogen atom and the formation of a covalent adduct (a thioether) between the nucleophilic cysteine and the inhibitor. Consistent with known Z-VAD-fmk-bound complexes [30, 31], the inhibitor molecule binds to the active site of MOA forming a thioether with the catalytic cysteine (Cys215). The electron density maps show well-defined, continuous electron density connecting S^γ^ of Cys215 and the C^1^ atom of the fluoromethyl group. The two atoms are placed at a distance of 1.7 Å, in good agreement with a covalent single C-S bond.

### Binding geometry of Z-VAD-fmk in the ‘direct’ orientation

**Fig 4:**
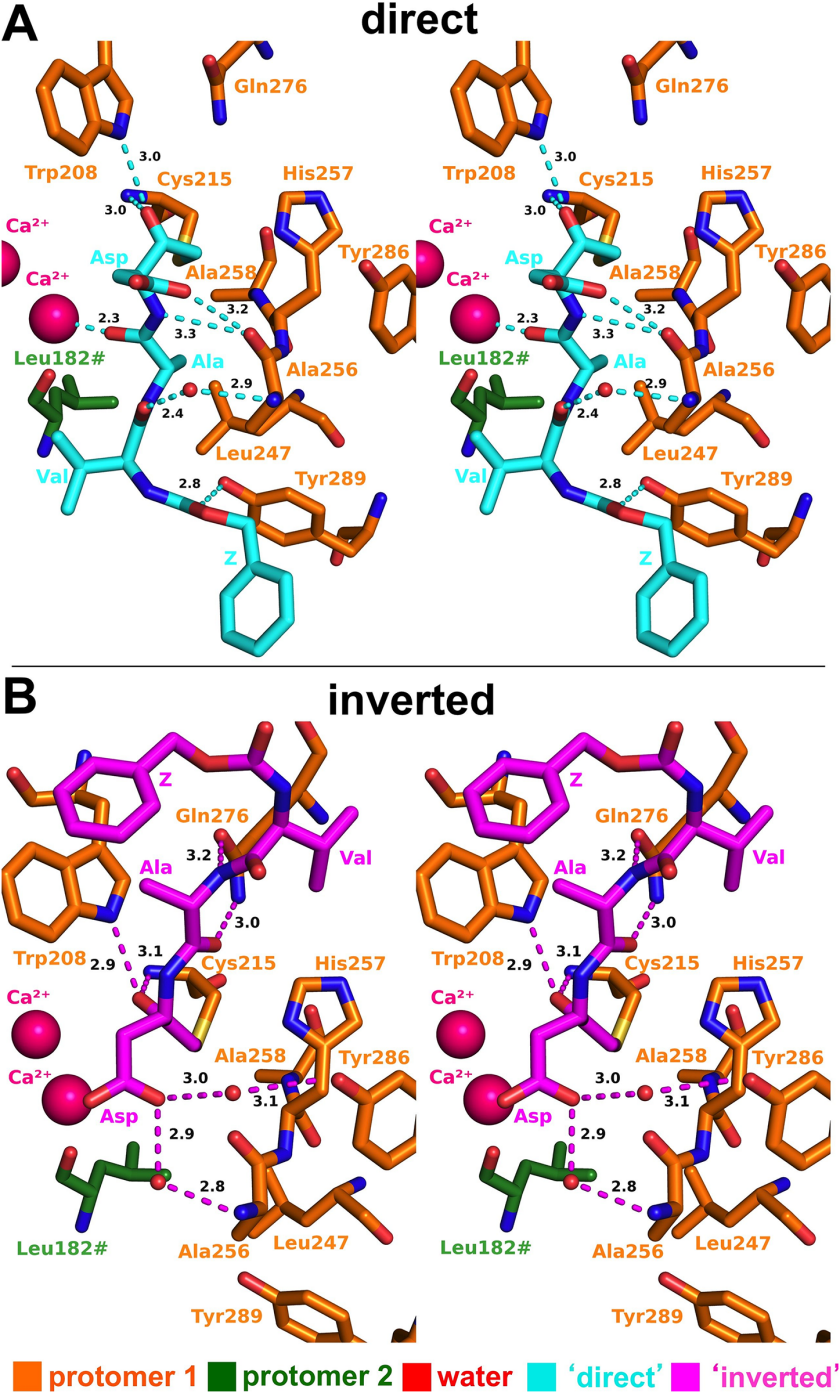
Z-VAD-fmk bound to the MOA active site. Stereographic representation of Z-VAD-fmk in (A) ‘direct’ (cyan) and (B) ‘inverted’ (magenta) orientations. Key interactions at the unprimed and primed subsites of the MOA catalytic cleft are depicted.

In the ‘direct’ orientation, the inhibitor molecule extends along the MOA dimerization interface, interacting with both protomers (Fig 3B). The carbonyl group of the aspartate residue lies within hydrogen bonding distance of the backbone NH group of the catalytic cysteine (3.0 Å) and the indole NH of Trp208 (3.0 Å; Fig 4A). The aspartate side chain of the inhibitor points away from the catalytic cleft, and forms a hydrogen bond with the backbone carbonyl oxygen of Ala256 (3.2 Å), which also engages in a hydrogen bonding interaction with the Asp backbone NH group (3.3 Å). The adjacent carbonyl group in the Ala-Asp peptide group interacts strongly with the solvent-exposed Ca^2+^ ion (2.3 Å), replacing a water molecule present in the calcium-bound structure of MOA.

Proceeding further along the chain, the inhibitor molecule interacts with residues provided by both protomers in the MOA dimer. The side chain of the alanine moiety of the VAD peptidyl group snugly fits into a hydrophobic cavity lined by residues Leu247, Ala258 and Leu182# (Fig 4A; ‘#’ denotes a residue provided by the symmetry-related protomer). The carbonyl group of the Val-Ala peptide group contacts the backbone NH of Ala256 through a water-mediated interaction (Z-VAD-fmk-Ala3(O)/HOH: 2.4 Å). The valine residue of Z-VAD-fmk is the last well-defined part of the inhibitor. Its side chain points towards a loop in the second protomer (Ile181#-Gly184#), approximately 6 Å away (distance to Ile181#). Beyond that point, the molecule is less ordered in its preferential orientation (S1B Fig), with the carbonyl moiety of the Z group interacting with the hydroxyl group of Tyr289 (2.8 Å; Fig 4A).

### Binding geometry of Z-VAD-fmk in the ‘inverted’ orientation

In the ‘inverted orientation’, Z-VAD-fmk interacts exclusively with one protomer (Figs 3C and 4B). Close to the newly formed covalent bond, the Z-VAD-fmk keto group interacts with both the backbone NH group of Cys215 (3.1 Å) and the indole NH of Trp208 (2.9 Å; Fig 4B). The side chain of the aspartate moiety points towards the solvent. Its carboxylate group superimposes well with its position in the ‘direct’ orientation, indirectly interacting with the enzyme through Ala256 (2.9 Å), and indirectly *via* two water molecules. The water molecules bridge the contact with the backbone NH group of Ala256 (Z-VAD-fmk-Asp4-O^δ1^/HOH: 2.9 Å) and the hydroxyl group of Tyr286 (Z-VAD-fmk-Asp4-O^δ1^/HOH: 3.0 Å), respectively.

The Ala-Asp peptide group of Z-VAD-fmk is oriented such that the NH group points towards the solvent, while the carbonyl group faces the catalytic cleft. This orientation results in a direct interaction with the side chain amide of Gln276 (Gln276-N^ε2^/Z-VAD-fmk-Ala3-O: 3.2 Å). The alanine side chain is projected towards the left side of the active site (standard view), facing the flat surface of the Trp208 side chain (Fig 4B). The carbonyl group of the Val-Ala peptide group points towards the solvent, while the peptidyl NH is directed towards the enzyme, engaging the oxygen atom of the Gln276 side chain (Gln276-O^ε1^/Z-VAD-fmk-Val2-N: 3.0 Å). The N-terminal end of the inhibitor interacts less strongly with MOA, pointing towards the Phe273-Gly279 loop.

### Deciphering the active site of MOA based on the homology with papain

**Fig 5:**
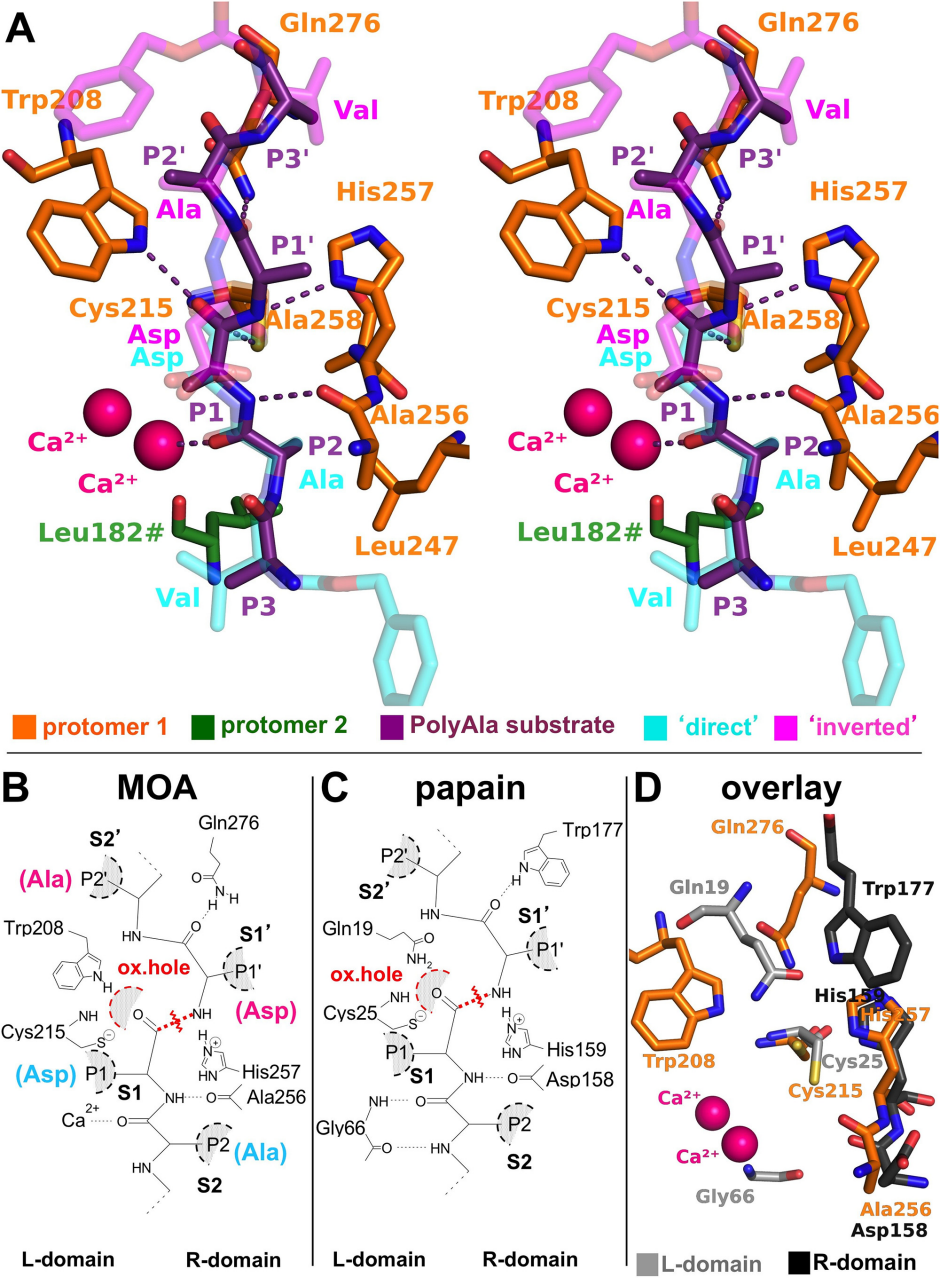
MOA-substrate interactions, derived according to the PLCP paradigm. (A) Stereographic representation of a manually docked polyalanine substrate (purple) to the active site of MOA. The peptide follows the PLCP substrate orientation and takes advantage of the interactions identified by the Z-VAD-fmk-MOA complex. (B,C) Side-by-side schematic representation of the substrate interactions derived for MOA (B) and papain (C); adapted from [5]). The oxyanion hole and the scissile bond are marked in red. (D) Structural superimposition of MOA (PDB ID: 3EF2 [14]) and papain (PDB ID: 1PPN [50]), revealing the Gly66-calcium substitution and the Trp-Gln swap.

**Fig 6:**
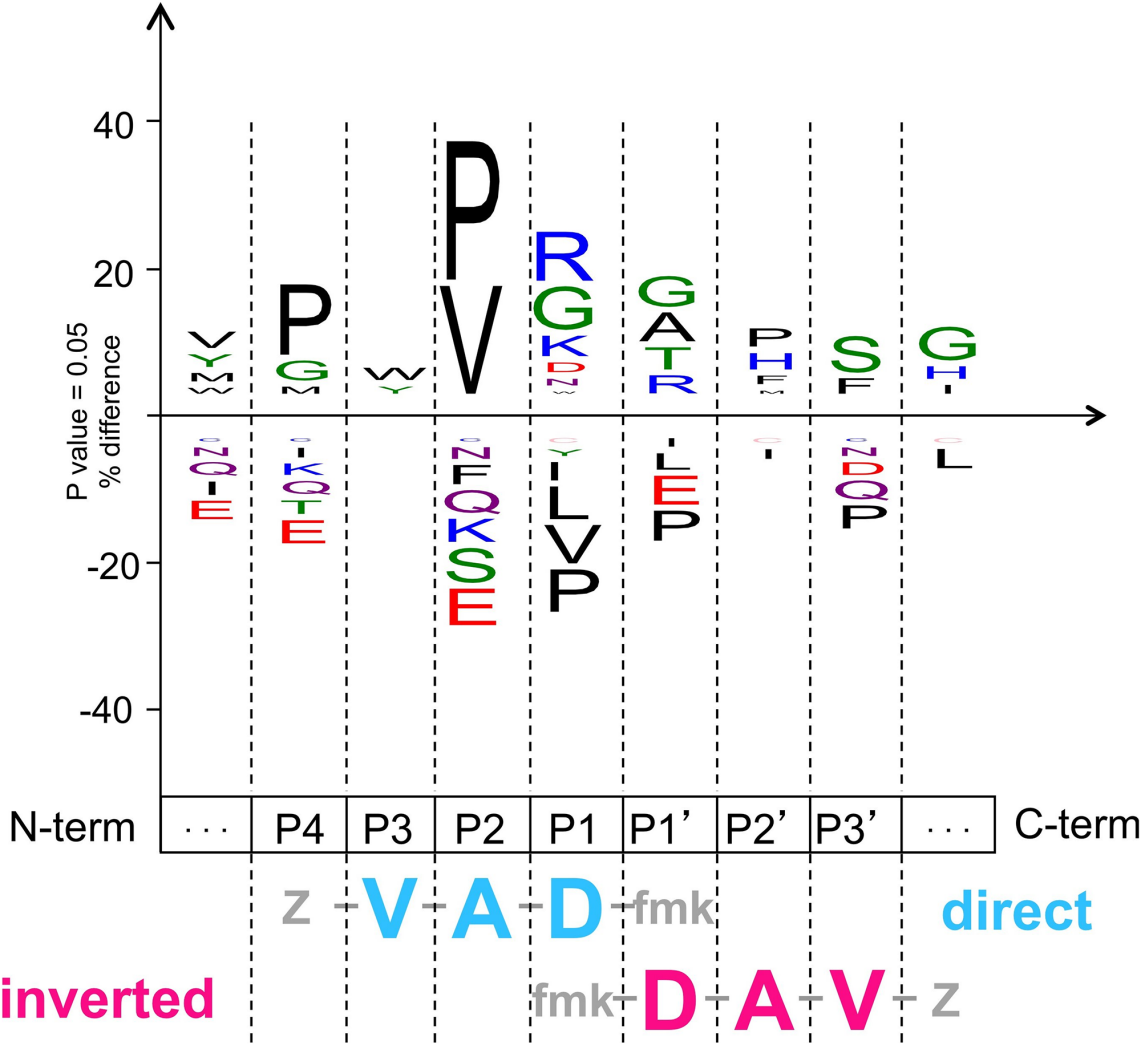
Position-dependent substrate preference of MOA. The diagram was generated *ex novo* through the iceLogo server (http://iomics.ugent.be/icelogoserver/logo.html) [51], using a database of peptides derived from the LC-MS analysis of the MOA proteolytic digestion products published by Wohlschlager *et al*. [18]. The Z-VAD-fmk peptide group in both the direct and inverted orientations is reported underneath the iceLogo diagram for a direct comparison with the binding preferences at each occupied subsite.

Overall, the inhibitor binding modes observed in the MOA-Z-VAD-fmk complexes are consistent with the PLCP paradigm. Based on the structural alignment with papain, Z-VAD-fmk occupies either the unprimed (S1-S3, ‘direct’ conformation) or the primed (S1’-S3’, ‘inverted’ conformation) subsites on the catalytic cleft of MOA (Fig 2). As for many PLCP-E-64 complexes [23, 46–49], the ‘inverted’ Z-VAD-fmk structure provides valuable insight into the fundamental enzyme-substrate interactions, in spite of its reverse backbone orientation. To ease interpretation, we extended the Z-VAD-fmk peptide chain from the ‘direct’ orientation through the electron density of the inhibitor in the ‘inverted’ orientation, which resulted in the substrate model shown in Fig 5A. The side-by-side analysis of the catalytic cleft of MOA and papain (Fig 5B, 5C and 5D) provides key pointers to important structural determinants for substrate recognition and catalysis. The combination of the papain-MOA structural alignment with the footprint of the Val-Ala-Asp side chains identifies the catalytic cleft regions corresponding to the S3-S3’ subsites of MOA, providing a rationale for the P3-P3’ substrate preference observed by Wohlschlager *et al*. [18] (Fig 6).

### Non-primed sites (S1-S3)

The non-primed sites (S1-S3) of MOA lie on the dimerization interface and receive contributions from both protomers (Fig 5A). The network of backbone-backbone interactions holding the substrate in place forces it into a conformation that has been likened to a strand in a very short β-sheet [52].

#### S1 subsite

The PLCP S1 subsite lies almost entirely on the L-domain. The most important and better conserved interactions take place between the carbonyl group of the scissile bond on the substrate and the ‘oxyanion hole’ on the enzyme (Fig 5B and 5C). This structural feature is a cavity surrounded by dipoles with the role of stabilizing the transition state during the proteolytic reaction [53, 54]. In PLCPs, the interaction is mediated by the backbone NH of the catalytic cysteine (Cys25 in papain; Fig 5C) and an electron-acceptor group from a nearby residue. The latter is usually provided by the side chain of a glutamine residue (Gln19 in papain), although some members of the papain superfamily have been shown to carry a different residue (*e*.*g*., a tyrosine residue in LapG). A second conserved interaction at the S1 position takes place between the backbone NH from the P1 residue of the substrate and the backbone carbonyl of the residue next to the catalytic His (Asp158 in papain).

In MOA, the latter interaction is conserved, involving the backbone carbonyl of Ala256, while the oxyanion hole exhibits a somewhat different structural framework (Fig 5B). Unlike papain, the NH group of the Trp208 indole ring replaces the glutamine-mediated interaction, whereas the backbone NH group of the catalytic cysteine (Cys215) represents a conserved feature. Gln19 variants of papain retain proteolytic activity [53–55]. This suggests that the oxyanion hole of PLCPs allows for a certain variability, and the conservation of the glutamine residue among family members is not of fundamental importance for the hydrolysis reaction.

Interestingly, the side chain of Asp158, originally thought to be the general acid-base catalyst in the hydrolytic reaction of papain [56] (a role fulfilled by His159), was later determined to play a marginal role in the thiolate-imidazolium pair stabilization [57]. An analysis of the reaction kinetics of different papain variants of Asp158 showed that, while non-essential, substitution of this amino acid with Ala decreased activity to 10% of the wild-type enzyme [58]. In MOA, the equivalent position is occupied by Ala256 (Fig 5B), which might contribute to its relatively low *in vitro* catalytic activity.

The substrate binding conformation in PLCPs forces the side chain of the P1 residue towards the solvent. This structural constraint limits the interaction with the enzyme, which is reflected by the lack of a stringent S1 substrate specificity for PLCPs. The binding surface for the P1 side chain in PLCPs is partly provided by a stretch of residues preceding the catalytic cysteine, and partly by residues from the neighboring Cys63-Gly66 loop. The S1 subsite of MOA is hinted at by the Z-VAD-fmk inhibitor aspartate moiety, which binds to the R-domain through water-mediated interactions. Based on PLCP paradigm, however, the S1 subsite of MOA extends to the L-domain, involving the side chain of Glu210, which would explain the preference for an arginine or a lysine residue at the P1 position (Fig 6).

#### S2 subsite

**Fig 7:**
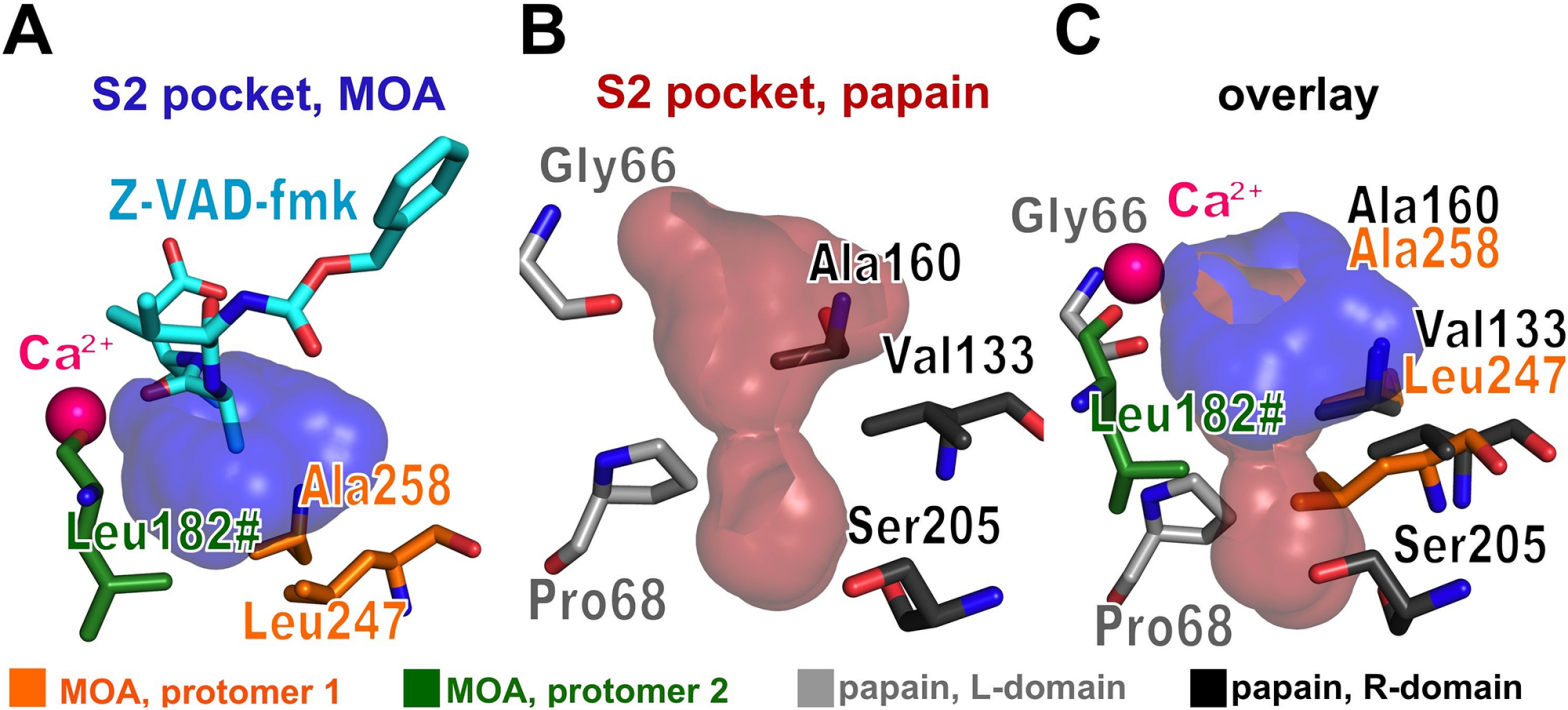
Substrate specificity pockets of MOA and papain. The left and center panels show the solvent-exposed surface at the specificity-determining S2 subsite of papain (A) or MOA (B). A structural alignment of the two proteins (panel C) shows a more shallow S2 binding pocket in MOA compared to papain, explaining the preference of MOA for small P2 residues.

The S2 binding site in PLCPs constitutes one of the main selectors for substrate specificity and, as such, is referred to as the ‘*substrate specificity pocket*’ [59, 60] (Figs 2 and 7). In papain, the substrate interacts with the enzyme through the backbone carbonyl and NH groups of a conserved glycine residue (Gly66), with the side chain of the P2 moiety pointing towards a cavity in the active site cleft. The cavity is lined by residues of the R- and the L-domain, the nature and size of which influence the physicochemical properties of the binding pocket. In papain, the walls of the cavity are formed by hydrophobic residues (Pro68, Val133, Ala160) (Fig 7), allowing the processing of substrates with an aliphatic side chain at the P2 position [61, 62]. The residue at the bottom of the cavity (Ser205 in papain) plays a key role in some PLCPs such as cathepsin B, conferring specificity for charged P2 residues, such as arginine [63].

The S2 binding site of MOA introduces two unique features among PLCPs: the presence of the calcium binding site and the dimerization interface (Fig 5A). The S2 specificity pocket of MOA is defined by the P2 alanine side chain of Z-VAD-fmk. Its walls and bottom are lined by the side chains of hydrophobic residues provided by both protomers (Ala258, Leu182# and Leu247) (Fig 7A), defining a more shallow cavity than its papain counterpart (Fig 7B and 7C). The shallow depth and the hydrophobic lining of the S2 pocket provide a structural rationale for the observed substrate preference for a proline or valine residue at the P2 position [18] (Fig 6). A single Ca^2+^ replaces the double backbone-mediated interaction of papain residue Gly66 (Fig 5B). Previous publications linked the calcium-induced opening of its catalytic cleft with the metal-dependence of MOA proteolytic activity [14, 22]. The direct involvement of one of the two metal ions in substrate coordination further reinforces this hypothesis, suggesting a functional involvement in the proteolytic reaction in addition to its structural role. The close distance between the calcium ion and the catalytic center suggests a possible influence of the metal ion on the kinetics of the enzymatic reaction; and in fact the presence (and nature) of the metal ion is essential for the catalytic reaction to occur [22]. The calcium ion is octahedrally coordinated [14] and can be functionally replaced by manganese (II) [22], and possibly other metal ions, with the potential of tuning the catalytic activity of MOA.

#### S3 subsite

According to the revised definition by Turk *et al*. [4], the S3 binding site primarily involves interactions with the side chains from an R-domain loop (His61-Ala67 in papain). Due to the low sequence conservation of the S3 binding region throughout the PLCP family, this site is usually referred to as ‘binding area’, in contrast to ‘subsite’, which is defined as a spatially well-conserved patch of residues among PLCPs. The corresponding structural feature of MOA is identified by the valine moiety of the Z-VAD-fmk inhibitor. It mainly corresponds to loop Ile181#-Gly184# (part of the L-domain), with a possible contribution from the neighboring Ser160#. The nature of the chemical groups exposed to the S3 subsite, mostly backbone carbonyls, suggests the preference for bulky residues carrying a polar group. A slight preference for tryptophan or tyrosine at the P3 position, as suggested by an analysis of proteolytic products (see iceLogo diagram in Fig 6), supports this notion.

### Primed sites (S1’- S3’)

In the primed sites, the backbone direction of the VAD tripeptide shows an inverted orientation compared to a PLCP substrate, and analogies are deduced more qualitatively. In the primed subsites of PLCPs, there is only one conserved contact point between the substrate backbone and the enzyme. This interaction involves the P1’ carbonyl group from the substrate backbone and the NH group of the indole ring of a conserved tryptophan residue (Trp177 in papain, Fig 5C). In contrast, substrate binding in MOA is mediated by the side chain amide group of a glutamine residue (Glu276). A structure-based sequence alignment of MOA with other papain-like cysteine proteases (not shown) reveals that the conserved glutamine and tryptophan residues in PLCPs (Gln19 and Trp177 in papain) are spatially and functionally swapped in MOA (Trp208 and Gln276) (Fig 5D). In PLCPs, Trp177 has been suggested to provide shielding of the thiolate-imidazolium pair from the solvent, but at the same time enhancing its nucleophilic character [64]. The much smaller footprint of the glutamine residue in MOA is expected to provide less shielding, consistent with its low enzymatic activity [18].

#### S1’ subsite

The S1’ binding site of PLCPs lies entirely on the R-domain. While the S1’ site is not a primary specificity selector, it contributes to the enzyme’s overall substrate preference [61]. In papain, the S1’ site is mostly formed by hydrophobic residues (Ala136, Ala137), with a hydrophilic component provided by two glutamine residues (Gln135, Gln142). Due to the distortion introduced by the inverted backbone orientation, the S1’ subsite surface of MOA is only hinted at by the Z-VAD-fmk aspartate side chain. While the terminal carboxylate group of the aspartate residue is engaged in water-mediated interactions with the backbone of Ala256 (Fig 4B), the actual S1’ binding surface likely involves a different group of residues (Fig 5A). The S1’ subsite provides a shallow groove, lined by the flat surface of the His257 imidazole ring on one side and by the hydroxyl group of an R-domain tyrosine residue (Tyr286) on the other. While the iceLogo diagram suggests glycine or alanine as the most favoured P1’ substrate moieties, Tyr286 provides a hydrophilic patch suitable for the interaction with a polar side chain; and indeed, the iceLogo diagram points to a minor preference for threonine or arginine at the P1’ position (Fig 6).

#### S2’ subsite

The S2’ binding area in PLCPs is identified by a loop capping the oxyanion hole, which directly precedes the catalytic cysteine (residues Gln19 to Ser24 in papain). The alanine moiety of Z-VAD-fmk projects towards MOA residue Trp208 (Fig 5A), which is part of a loop considerably shorter than its papain counterpart. The tryptophan indole ring provides a broad, hydrophobic surface area to interact with the P2’ side chain, which could limit the P2’ moieties to amino acids with low steric hindrance or alternatively serve as a platform for aromatic stacking interactions. The iceLogo diagram suggests a weak preference for proline, histidine, phenylalanine or methionine as P2’ residues (Fig 6), representing both shorter residues similar to the Ala in Z-VAD-fmk, and candidates for stacking.

#### S3’ subsite

Binding sites beyond positions S3’ or S2’ are not universally defined in PLCPs, as the interaction surface varies for each member, requiring a case-by-case investigation. The valine moiety of the ‘inverted’ Z-VAD-fmk inhibitor points to an interaction site in the R-domain. While the predicted S3’ binding area is lined by the hydrophilic residues Glu274, Gln276 and Asn277, the carboxylate of Glu274 points away from the Val of Z-VAD-fmk, exposing the hydrophobic part of its side chain. Other possible hydrophobic contributions are provided by the neighboring residues Leu281 and Ile284. This site could potentially accommodate a variety of residues with different properties. The iceLogo diagram shown in Fig 6), points to a weak preference for Ser or Phe at the P3’ position.

## Conclusions

The dimerization domain of MOA represents a novel embodiment of the papain fold with peculiar characteristics, which set it apart from known papain-like proteases. The unique features of MOA include the presence of a binuclear calcium-binding site and a substrate binding cleft contributed by residues from both protomers. The sharing of the binding cleft between the two protomers hints at a functional coupling, which extends beyond their mutual structural support. Another important feature exposed by the MOA Z-VAD-fmk structure is a tryptophan-glutamine structural and functional swap and the direct involvement of one of the metal ions in substrate interaction. While previous data already pointed to the importance of the calcium-induced conformational change in enzyme activation, the direct interaction between one of the calcium ions and the proteolytic substrate implies additional roles of metal binding in regulating the enzymatic activity. Further structural and biochemical investigations on MOA and homologues are required to explore this possibility and confirm the catalytic role of each active site element, in order to shed further light on the inner workings of this unusual addition to the clan CA cysteine proteases.

## Supporting Information

S1 Fig Stereographic representation of ligand electron density. (A,B) ‘ZVAD-direct’, (C,D) ‘ZVAD-inverted’ and (E,F) ‘ZVAD-dual’ structures. On the left side, panels A, C and E show the final σ_A_-weighted 2m*F*o-D*F*c map (grey, contoured at 1σ) calculated for the coordinates of the Z-VAD-fmk molecule and the catalytic cysteine (Cys215). On the right side, in panels B, D, and F, the σ_A_-weighted m*F*o-D*F*c difference density map of the same region before the inclusion of the Z-VAD-fmk ligand is shown for comparison (green, contoured at 3σ). Partial occupancy for Asp in the alternative orientation is noticeable in the ‘ZVAD-direct’ and the ‘ZVAD-inverted’ structures when a lower sigma cut-off is applied.
(TIF)
