# Supplementary figures and images for: An Unusual Member of the Papain Superfamily: Mapping the Catalytic Cleft of the Marasmius oreades agglutinin (MOA) with a Caspase Inhibitor
Source: PLoS One. 2016 Feb 22;11(2):e0149407. doi: 10.1371/journal.pone.0149407 (PMC4764322; doi:10.1371/journal.pone.0149407)

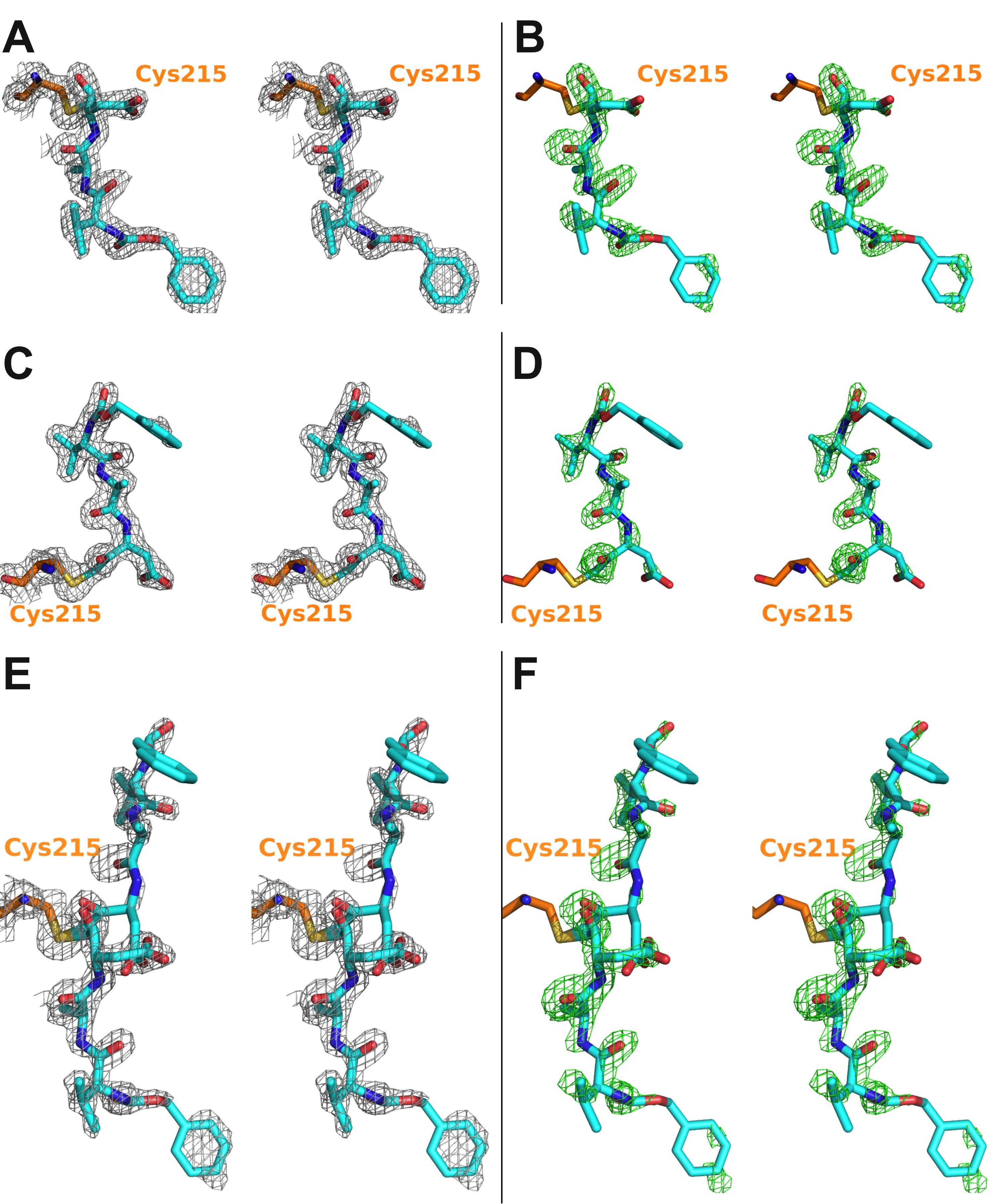

Supplement: S1 Fig — (A,B) ‘ZVAD-direct’, (C,D) ‘ZVAD-inverted’ and (E,F) ‘ZVAD-dual’ structures. On the left side, panels A, C and E show the final σA-weighted 2mFo-DFc map (grey, contoured at 1σ) calculated for the coordinates of the Z-VAD-fmk molecule and the catalytic cysteine (Cys215). On the right side, in panels B, D, and F, the σA-weighted mFo-DFc difference density map of the same region before the inclusion of the Z-VAD-fmk ligand is shown for comparison (green, contoured at 3σ). Partial occupancy for Asp in the alternative orientation is noticeable in the ‘ZVAD-direct’ and the ‘ZVAD-inverted’ structures when a lower sigma cut-off is applied. (TIF) [file pone.0149407.s001.TIF]
